# Reported Neighborhood Traffic and the Odds of Asthma/Asthma-Like Symptoms: A Cross-Sectional Analysis of a Multi-Racial Cohort of Children

**DOI:** 10.3390/ijerph18010243

**Published:** 2020-12-31

**Authors:** Sarah Commodore, Pamela L. Ferguson, Brian Neelon, Roger Newman, William Grobman, Alan Tita, John Pearce, Michael S. Bloom, Erik Svendsen, James Roberts, Daniel Skupski, Anthony Sciscione, Kristy Palomares, Rachel Miller, Ronald Wapner, John E. Vena, Kelly J. Hunt

**Affiliations:** 1 Department of Environmental and Occupational Health, Indiana University, Bloomington, IN 47405, USA; 2 Department of Public Health Sciences, Medical University of South Carolina, Charleston, SC 29425, USA; ferguspl@musc.edu (P.L.F.); neelon@musc.edu (B.N.); pearcejo@musc.edu (J.P.); svendsee@musc.edu (E.S.); Vena@musc.edu (J.E.V.); huntke@musc.edu (K.J.H.); 3 Department of Obstetrics and Gynecology, Medical University of South Carolina, Charleston, SC 29425, USA; newmanr@musc.edu; 4 Department of Obstetrics and Gynecology, Northwestern University, Chicago, IL 60611, USA; w-grobman@northwestern.edu; 5 Department of Obstetrics and Gynecology, University of Alabama at Birmingham, Birmingham, AL 35233, USA; atita@uabmc.edu; 6 Department of Global and Community Health, George Mason University, Fairfax, VA 22030, USA; mbloom22@gmu.edu; 7 Department of Pediatrics, Medical University of South Carolina, Charleston, SC 29425, USA; robertsj@musc.edu; 8 Department of Obstetrics and Gynecology, New York-Presbyterian Queens Hospital, Queens, NY 11365, USA; dwskupsk@med.cornell.edu; 9 Department of Obstetrics and Gynecology, Weill Cornell Graduate School of Medical Sciences, Cornell University, New York, NY 10021, USA; 10 Department of Obstetrics and Gynecology, Christiana Care Health System, Wilmington, DE 19899, USA; ASciscione@ChristianaCare.org; 11 Department of Obstetrics and Gynecology, Saint Peter’s University Hospital, New Brunswick, NJ 08901, USA; kpalomares@saintpetersuh.com; 12 Department of Medicine, Division of Clinical Immunology, Icahn School of Medicine at Mount Sinai, New York, NY 10029, USA; rachel.miller2@mssm.edu; 13 Columbia University Irving Medical Center, Department of Obstetrics and Gynecology, Columbia University, New York, NY 10032, USA; rw2191@cumc.columbia.edu

**Keywords:** air pollution exposure, asthma, racial/ethnic disparities, neighborhood traffic, cohort

## Abstract

Asthma in children poses a significant clinical and public health burden. We examined the association between reported neighborhood traffic (a proxy for traffic-related air pollution) and asthma among 855 multi-racial children aged 4–8 years old who participated in the Environmental Influences on Child Health Outcomes (ECHO) cohort. We hypothesized that high neighborhood traffic density would be associated with the prevalence of asthma. Asthma/asthma-like symptoms (defined as current and/or past physician diagnosed asthma, past wheezing, or nighttime cough or wheezing in the past 12 months) was assessed by parental report. The relationship between neighborhood traffic and asthma/asthma-like symptoms was assessed using logistic regression. The prevalence of asthma/asthma-like symptoms among study participants was 23%, and 15% had high neighborhood traffic. Children with significant neighborhood traffic had a higher odds of having asthma/asthma-like symptoms than children without neighborhood traffic [adjusted OR = 2.01 (95% CI: 1.12, 3.62)] after controlling for child’s race-ethnicity, age, sex, maternal education, family history of asthma, play equipment in the home environment, public parks, obesity and prescribed asthma medication. Further characterization of neighborhood traffic is needed since many children live near high traffic zones and significant racial/ethnic disparities exist.

## 1. Introduction

Asthma is a common chronic disease with varying clinical and biological characteristics [1,2,3]. The disease is associated with increased morbidity, mortality, increased health care costs and parental work and children’s school absence [4,5]. In the United States, asthma affects approximately 9.3% of children and this translates to a large clinical and economic burden on the country’s health care system [6,7,8,9]. A recent study reported that asthma-related costs were approximately $80 billion in 2013 when factors such as missed school/work days, medical costs and mortality were combined [10].

The interplay between environmental and genetic factors adds to the complexity of asthma. Exposure to environmental factors, such as air pollution, may increase the prevalence of asthma and other adverse respiratory symptoms [11,12,13,14,15,16]. Emerging evidence also shows that residential proximity to a major roadway and exposure to traffic-related air pollution (TRAP) during childhood is associated with the onset of asthma [17,18,19] as well as acute airway inflammation in individuals with and without asthma [20,21,22,23,24,25].

There is mounting evidence that living near heavy traffic is associated with increased rates of asthma, cardiovascular disease, and dementia [26,27,28,29,30]. Additionally, air pollution exposure gradients at small scales such as neighborhoods are associated with adverse health effects [31,32]. Although direct assessment of individual TRAP exposure is ideal, it presents a myriad of logistical challenges in population-based studies. Simple proxies, such as distance to roadways and traffic estimates or counts, can also be used to assign individual TRAP exposure [26,32,33,34,35,36,37,38,39,40,41].

Economically disadvantaged and minority populations share a disproportionate burden of air pollution exposure and risk [42]. Growing evidence shows that these populations experience higher residential exposure to traffic and TRAP than non-minorities and persons of higher socioeconomic status [43,44]. Children are more susceptible to air pollution exposures than adults due to immature immune systems, developing lungs, higher breathing rates, a greater extent of mouth breathing and more outdoor activities [12,45,46].

Racially diverse cohort studies can provide further evidence and advance the state of the science on the relationships between asthma and air pollution. A recent international workshop highlighted the need to clarify modes of asthma progression and exacerbations during pre-puberty and the relevance of environmental exposures [47]. With the presence of a ‘triple jeopardy’, namely the combination of impaired respiratory health, racial/ethnic status, and TRAP exposures, it is essential to identify subgroups of the population that may need to be targeted for intervention. Accordingly, we used parent-reported density of neighborhood traffic as a proxy measure for TRAP and evaluated its association with the prevalence of reported asthma/asthma-like symptoms in children 4 to 8 years old, who participated in the Environmental Influences on Child Health Outcomes (ECHO) cohort (*n* = 855). Our main hypothesis was that increased/high neighborhood traffic density would be associated with the prevalence of asthma in our study cohort. To test this hypothesis in this racially and geographically diverse cohort, we sought to determine what proportion of children were reported to have high neighborhood traffic and whether this exposure was associated with asthma/asthma-like symptoms.

## 2. Materials and Methods

### 2.1. Study Population

This study leveraged a unique resource from the *Eunice Kennedy Shriver* National Institute of Child Health and Human Development Fetal Growth Studies (NICHD FGS). Specifically, a cohort of racially, ethnically and geographically diverse healthy women and fetuses were studied longitudinally across twelve sites in the United States during pregnancy from July 2009 to January 2013, to characterize optimal fetal growth velocity [48].

The ECHO study was designed and implemented to follow up with the NICHD FGS cohort. There were 1116 mother singleton child pairs recruited from May 2017 through April 2019 for ECHO. Ten of the original twelve NICHD FGS study sites participated in ECHO: Christiana Care Health System (DE), Columbia University Irving Medical Center (NY), Fountain Valley Regional Hospital and Medical Center (CA), Medical University of South Carolina (SC), Miller Children’s Hospital Long Beach Memorial Medical Center (CA), New York Hospital Queens (NY), Northwestern University Feinberg School of Medicine (IL), St. Peter’s University Hospital (NJ), University of Alabama at Birmingham (AL), and University of California at Irvine (CA).

Mothers were invited to participate in self-report surveys while physical and neurodevelopmental data were collected from children. If a mother–child pair was unable or unwilling to attend an in-person visit, they could still participate in a home visit, telephone visit or a short computer survey. Participants were remunerated for their time, depending on the type of visit completed. Written informed consent was obtained from the parent or legal guardian of each enrolled child and depending on child age and state regulations child assent was obtained when required. We used Research Electronic Data Capture (REDCap) to enter data from paper forms as needed, to allow electronic form data to be directly captured, and to securely store data.

The REDCap electronic data capture tool is hosted by the South Carolina Clinical and Translational Science (SCTR) Institute at the Medical University of South Carolina. It is a secure, web-based software platform designed to support data capture for research studies, providing (1) an intuitive interface for validated data capture; (2) audit trails for tracking data manipulation and export procedures; (3) automated export procedures for seamless data downloads to common statistical packages; and (4) procedures for data integration and interoperability with external sources.

REDCap at SCTR is supported by the National Center for Advancing Translational Sciences of the National Institutes of Health under Grant Number UL1 TR001450.

During recruitment, parents received detailed information regarding the purposes of the project and were given the opportunity to voice any concerns and/or to sign the informed consent. This study utilized a central IRB (cIRB) through Columbia University for all ten participating sites. A SMART IRB Master Common Reciprocal Institutional Review Board Authorization Agreement (“SMART IRB Agreement”) was used to establish reliance between the participating sites and the Columbia University IRB. This study was conducted in accordance with the standards of Good Clinical Practice. IRB approval numbers: IRB-AAAR4976, IRB-AAAR5769. Written informed consent was obtained from the parents or legal guardians of each enrolled child.

### 2.2. Questionnaires

Validated questionnaires included the Preschool-aged Children’s Physical Activity Questionnaire (Pre-PAQ) [49] and the International Study of Asthma and Allergies in Childhood (ISAAC) [50] questionnaire. Detailed questions were asked on child and family history of disease including asthma/asthma-like symptoms as well as questions related to demographic characteristics. The adult participant (99% were the mother) filled out these surveys either at home or during a study visit, with site personnel acting as translators as needed.

### 2.3. Exposure Variables

Primary Exposures: Reported current exposure to neighborhood traffic defined by the question, “There is so much traffic along the streets that it makes it difficult or dangerous to walk with my child in my neighborhood.” This statement is from a set of eight found in the Pre-PAQ (Q22). Children were considered exposed to significant neighborhood traffic when their mothers responded “Agree” or “Strongly Agree” to the above question. Accordingly, when mothers responded “Disagree” or “Strongly disagree”, those children were considered unexposed to significant neighborhood traffic.

Secondary Exposures: We included maternal response to questions on the home environment and neighborhood to assess other environmental factors that could influence a child’s exposure to TRAP. First, we examined the remaining seven questions in the Pre-PAQ (Q22) on neighborhood, with the same four Likert scale responses as above: (i) safety when playing outdoors; (ii) presence of local footpaths; (iii) barriers or dangers to walking; (iv) sufficient traffic lights or pedestrian crossings; (v) unsafe levels of crime; (vi) shops within walking distance; and vii) dangers in local parks. Then, for the home environment, we looked at access to any of the following facilities within the backyard or home environment (Pre-PAQ Q18): (i) play equipment (e.g., swing set, slide, climbing gym); (ii) pool or spa; and (iii) area suitable to ride a tricycle or scooter. Thirdly, for additional neighborhood characteristics, we evaluated maternal response to questions on whether a child’s local neighborhood had the following places or facilities where he/she can play and be physically active (Pre-PAQ Q21): (i) open areas such as beaches, rivers, natural reserves; (ii) public park or oval; iii) playground; (iv) public swimming pool; (v) gym that offers programs for young children (e.g., kindergym, playgym); and (vi) club that offers activities/sports for young children (e.g., soccer, dance).

### 2.4. Outcome Variable

**Figure 1:**
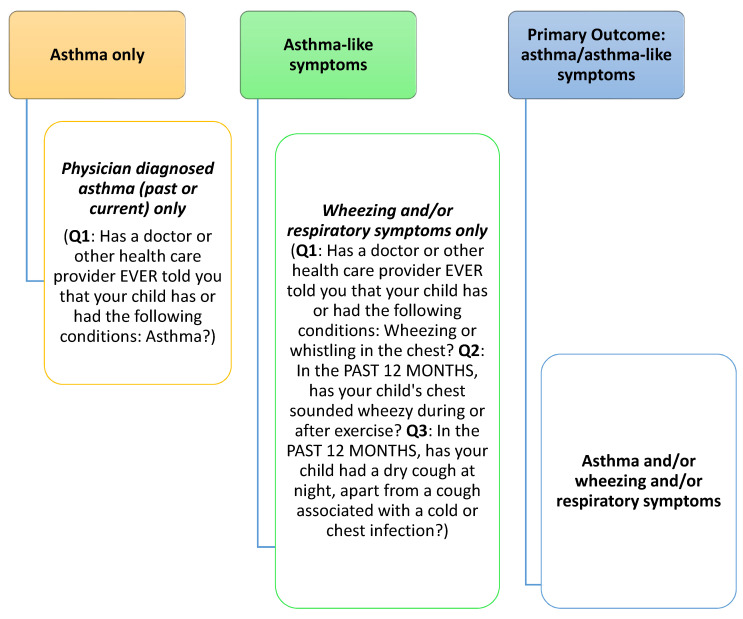
Definition of primary outcome: asthma/asthma-like symptoms.

The presence of wheezing, cough, and/or asthma was assessed by parental report using the validated ISAAC questionnaire. Asthma was considered positive when mothers responded “yes” to the question: “Has a doctor or other health care provider ever told you that your child has or had the following condition: Asthma?”. Wheezing/cough symptoms were considered positive when mothers responded “yes” to any of the following questions: Q1: “Has a doctor or other health care provider ever told you that your child has or had the following conditions: Wheezing or whistling in the chest?”; Q2: “In the past 12 months, has your child’s chest sounded wheezy during or after exercise?”; Q3: “In the past 12 months, has your child had a dry cough at night, apart from a cough associated with a cold or chest infection?”. In this analysis, we combine asthma, cough, and/or wheezing symptoms as *one primary outcome* termed: asthma/asthma-like symptoms (Outcome 3 in Figure 1). The definition of our primary outcome is based on previous ISAAC analyses, which have reported that a combination of these questions correlated more closely with asthma mortality and the overall definition of asthma-like symptoms than current wheeze alone [51].

### 2.5. Covariates

For adjusted analyses, we include the following the covariates into the model, as these have been identified as potential confounders in the literature: (1) child’s sex, (2) child’s race-ethnic group, (3) positive family history of asthma (asthma diagnosed in parents, siblings and/or grandparents), (4) maternal education level (less than or up to high school or greater than high school, (5) obese status of child, defined as having a BMI-for-age ≥95th percentile [52,53]. Further, we looked at other covariates such as presence of cats or dogs during child’s first year and/or within the past 12 months, secondhand/environmental tobacco smoke exposure, as well as responses to 16 questions from the Pre-PAQ (these described the home and neighborhood characteristics, such as the presence of parks, pools, shops, and street safety). Finally, we also assessed these factors in final models in our evaluation of the relationship to neighborhood traffic and asthma and asthma-like symptoms: (i) prescribed asthma medication (Inhalers and other asthma drug prescriptions (e.g., Pulmicort, Flovent, Singulair, Zyflo, Orapred, Advair, Symbicort, or Xolair), (ii) current residence in an area defined as an urban or rural census tract, (iii) respiratory allergy (defined by the question: Has a doctor or other health care provider ever told you that your child has or had hay fever or respiratory allergy (to pets, pollen, mold, dust, mites, etc.?).

### 2.6. Statistical Analysis

The primary aims of this study were to determine what proportion of children had high neighborhood traffic and whether this exposure was associated with asthma/asthma-like symptoms. We examined differences in children with or without asthma/asthma-like symptoms (and with or without significant neighborhood traffic) using χ^2^ tests for categorical variables and two-sample t-tests for continuous variables.

Next, we conducted a series of logistic regressions to evaluate the association between reported neighborhood traffic exposure and the occurrence of any reported asthma/asthma-like symptoms in the cohort. Model 1 included neighborhood traffic as the primary variable, and demographics as covariates. Models 2 and 3 incorporated additional covariates related to outdoor air exposure found to be significantly related to asthma/asthma-like symptoms. Models 4 and 5 included all statistically significant covariates and with the addition of child’s obesity status (model 4) and prescribed asthma medication (model 5) to determine whether these factors affected the final model.

We also examined the impact of including adjustment for factors such as mode of delivery (vaginal or caesarian section), child’s use of antibiotic medication in the past year, child’s stay at the neonatal intensive care unit after birth and gestational age at delivery. Lastly, we stratified the analysis by sex. To check the robustness of our results, we conducted a sensitivity analysis by limiting the primary health outcome to current asthma and/or current asthma-like symptoms as opposed to current or past asthma/asthma-like symptoms. We report results in terms of *p*-values, adjusted odds ratios (OR) and 95% confidence intervals. All statistical tests were two tailed, and *p*  ≤  0.05 was considered statistically significant. Analyses were conducted using SAS version 9.4 (SAS Institute Inc., Cary, NC, USA). The odds ratios plot was derived using ‘ggplot2′ function in R [54].

## 3. Results

### Overview

**Figure 2:**
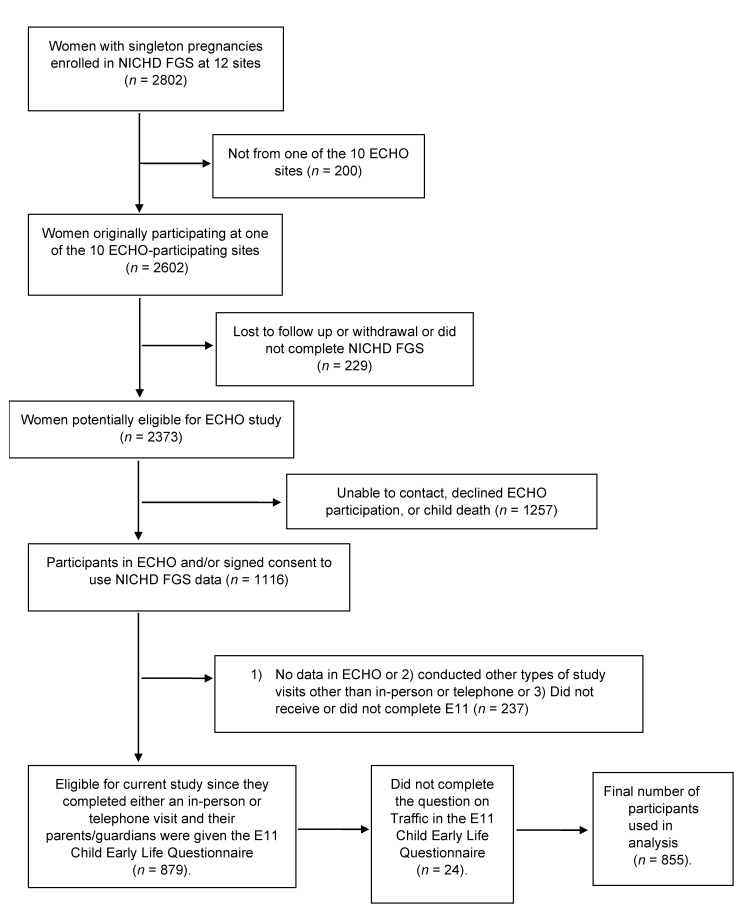
Sample size used in the present study in relation to the original Eunice Kennedy Shriver (Child Health and Human Development) NICHD Fetal Growth Studies cohort. Environmental Influences on Child Health Outcomes (ECHO).

For this study, 1116 children aged 4 to 8 years, born while their mothers participated in the NICHD-FGS study, were enrolled with their mothers in ECHO. Mothers of 879 children completed the study’s Child Early Life Questionnaire. However, 2.7% (*n* = 24) were excluded from the statistical analysis due to a lack of response to the question on neighborhood traffic (Figure 2). Hence, the current analysis includes 855 children. The sociodemographic and neighborhood characteristics of participants according to maternal report is provided for the exposure and outcome variables of interest—neighborhood traffic (Table 1) and asthma/asthma-like symptoms (Table 2), respectively. The prevalence of maternal report of doctor-diagnosed asthma was 13%; wheezing symptoms, including nighttime cough was 19%; and asthma and/or wheezing was 23% (hereafter asthma/asthma-like symptoms) (Table 1).

- Differences by race-ethnic group

Children of Hispanic (34.9%) and non-Hispanic Black (39.5%) race/ethnicity were more likely to be exposed to significant neighborhood traffic than non-Hispanic White children (12.4%) (*p*-value < 0.0001) (Table 1). Overall, mothers reported that 15.1% (*n* = 129) of the children in this analysis were exposed to significant neighborhood traffic (Table 2). Hispanic (31.5%) and non-Hispanic Black (40.1%) children were more likely to report asthma/asthma-like symptoms than non-Hispanic White or Asian children (*p*-value < 0.0001) (Table 2).

- Bivariate analysis—neighborhood traffic

Children exposed to neighborhood traffic were similar in terms of male sex, age, family history of asthma, obese status, presence of cats or dogs during child’s first year and/or within the past 12 months (*p* > 0.1) when compared to those unexposed to neighborhood traffic (Table 1). Walking distance to local shops, having a pool or spa in the backyard and access to public swimming pool were also not significantly different among the two neighborhood traffic groups. Maternal education, race, asthma, and wheezing symptoms as well as responses to a majority of the neighborhood factors (13/16 questions from the Pre-PAQ) differed significantly depending on reported neighborhood traffic (*p* < 0.05, Table 1). Secondhand smoke exposure showed marginal significance with increased neighborhood traffic (*p* = 0.08), and children with any household pet (dog and/or cat) were less likely to be exposed to neighborhood traffic (*p* = 0.02) but after adjusting for demographics and family history of asthma this was no longer statistically significant.

- Bivariate analysis—asthma/asthma-like symptoms

As shown in Table 2, the following factors were more likely to occur among children with asthma/asthma-like symptoms than in those without such symptoms: Male sex (56.9% vs. 49.5%, *p* = 0.07), non-Hispanic Black (40.1% vs. 26.6%) or Hispanic (31.5% vs. 24.2%) [*p* < 0.0001], family history of asthma (55.8% vs. 26.8%, *p*  <  0.0001), obese status, (21.3% vs. 8.2%, *p*  <  0.001), child’s exposure to second hand smoke (15.7% vs. 9.6%, *p*  = 0.02), neighborhood traffic (21.8% vs. 13.1%, *p*  =  0.003), dangers in the local parks (e.g., unleashed dogs or undesirable people) [13.2% vs. 8.2%, *p*  =  0.04], and the presence of a public park or oval in the local neighborhood for the child to play and be physically active (90.4% vs. 86.0%, *p*  =  0.03). On the other hand, having usable footpaths on most of the streets in local area (72.6% vs. 79.6%, *p*  =  0.04) and sufficient traffic lights or pedestrian crossings to make it safe to walk around neighborhood (71.6% vs. 78.9%, *p*  =  0.06) were reported less frequently by mothers of children with asthma/asthma-like symptoms than in those without such symptoms.

- Multiple logistic regression models

**Figure 3:**
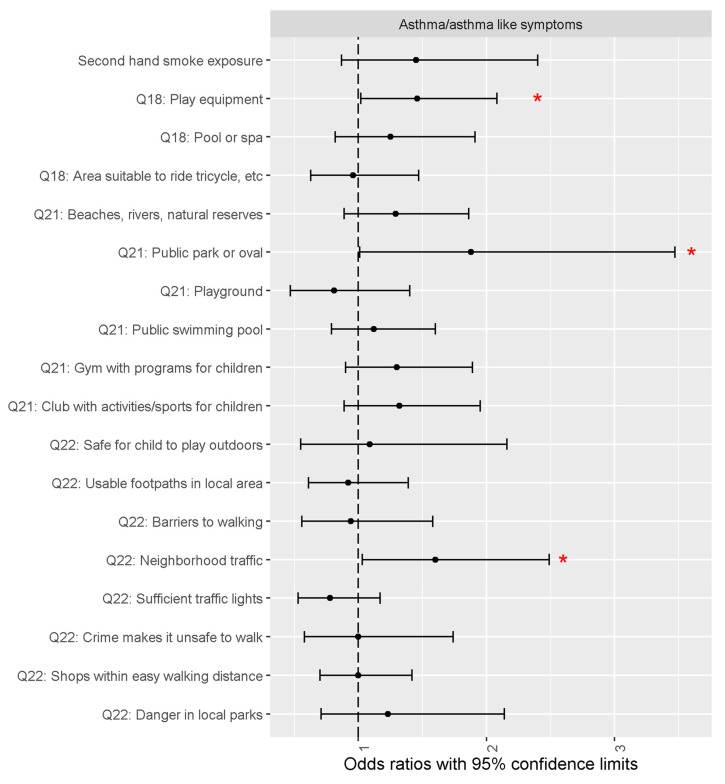
Odds ratios estimates with 95% confidence limits for individual covariates of interest for asthma/asthma-like symptoms, including secondhand smoke exposure as well as each of the Pre-PAQ Q18, Q21 and Q22 variables. Each covariate was in a separate logistic regression model, and the OR estimates are adjusted for child sex, child age, race-ethnic group, mom education and family history of asthma. Red asterisk (*) indicates statistically significant result.

In demographic-adjusted logistic regression models (i.e., models 1–3), neighborhood traffic, the presence of play equipment in the home environment or backyard, and the presence of a public park or oval in the neighborhood were positively associated with asthma/asthma-like symptoms (Figure 3). There were positive associations between asthma/asthma-like symptoms and the following groups: (1) males, (2) Non-Hispanic Blacks, (3) Hispanics, and (4) those with a family history of asthma (Table 3).

Model 4 in Table 3 (*n* = 738), revealed that children exposed to significant neighborhood traffic had higher odds of having asthma/asthma-like symptoms than children exposed to less neighborhood traffic [OR = 1.78 (95% CI: 1.10, 2.88)]. Obese children and children with play equipment in the home environment or backyard had higher odds of having asthma/asthma-like symptoms than those without: [OR = 1.60 (95% CI: 1.09, 2.35)] and [OR = 2.54 (95% CI: 1.55, 4.17)], respectively. Adding obesity status to the model resulted in the presence of a public park becoming non-significant. Non-Hispanic Black and Hispanic children had higher odds of having asthma/asthma-like symptoms than non-Hispanic White children: [OR = 2.53 (95% CI: 1.43, 4.50)] and [OR = 2.25 (95% CI: 1.28, 3.94)] respectively. Additionally, male children had higher odds of asthma/asthma-like symptoms [OR = 1.53 (95% CI: 1.06, 2.22)] (Table 3), as did children with a family history of asthma [OR = 3.19 (95% CI: 2.20, 4.64)]. The final model adjusted for prescribed asthma medication and reported neighborhood traffic remained statistically significant: OR = 2.01 (95% CI: 1.12, 3.62, model 5, *n* = 733). Male sex lost statistical significance and prescribed asthma medication was associated with the highest odds of reported asthma/asthma-like symptoms: OR = 25.07 (95% CI: 14.81, 42.45).

In a stratification analysis, children who had been prescribed asthma medication had a statistically significant increased odds of asthma/asthma-like symptoms (OR = 2.07 (95% CI: 1.11, 3.87; *n* = 613), compared to those who had not been prescribed asthma medication (OR = 1.01 (95% CI: 0.30, 3.39; *n* = 138). Additionally, when the results from model 5 are stratified by current rural vs. urban residence, those children currently residing in areas designated as urban tracts, had significantly increased asthma/asthma-like symptoms (OR = 1.98 (95% CI: 1.09, 3.59; *n* = 677) compared those residing in rural areas (OR = 2.16 (95% CI: 0.52, 9.01; *n* = 110) after adjusting for child’s race-ethnicity, age, sex, maternal education, family history of asthma, play equipment in the home environment, public parks, obesity and having prescribed asthma medication.

- Sensitivity analysis

When we limited the primary health outcome to current asthma and/or current wheezing (19%) as opposed to current or past asthma/asthma-like symptoms (23%), the results were similar. The only difference was that play equipment in the backyard was no longer statistically significant [OR = 1.54 (95% CI: 0.94, 2.51)]. Mother’s education [OR = 1.07 (95% CI: 0.62, 1.87)], Asian race [OR = 1.29 (95% CI: 0.47, 3.51)], male sex [OR = 1.37 (95% CI: 0.86, 2.18)] and the presence of neighborhood parks [OR = 2.19 (95% CI: 0.94, 5.07)] did not reach statistical significance. All other variables remained statistically significant: non-Hispanic Black [OR = 2.90 (95% CI: 1.38, 6.11)]; Hispanic [OR = 2.88 (95% CI: 1.40, 5.91)]; family history of asthma [OR = 2.15 (95% CI: 1.34, 3.45)]; obese [OR = 2.21 (95% CI: 1.19, 4.12)]; prescribed asthma medication [OR = 18.33 (95% CI: 11.11, 30.51)] and significant neighborhood traffic [OR = 2.07 (95% CI: 1.14, 3.77)]. When the final model was stratified by sex, there was no association between neighborhood traffic and asthma/asthma-like symptoms among females: OR = 1.23 (95% CI: 0.48, 3.13) [*n* = 358]. However, the males (*n* = 375) had a statistically significant association [OR = 2.95 (95% CI: 1.34, 6.46)].

- Other variables independently associated with asthma/asthma-like symptoms

We examined factors such as mode of delivery (vaginal or caesarian section), child’s use of antibiotic medication in the past year, child’s stay at the neonatal intensive care unit after birth and gestational age at delivery. Gestational age at delivery (range 30.1 to 42.7 weeks) was positively associated with reported asthma/asthma-like symptoms (OR: 1.27; 95% CI: 1.08, 1.49) after controlling for all variables in the final model (model 5). Reported neighborhood traffic remained statistically significant in this model as well (OR: 2.06; 95%CI: 1.13, 3.75).

## 4. Discussion

In this study, we sought to determine the prevalence of reported neighborhood traffic (a proxy for TRAP exposure) and its association with childhood asthma/asthma-like symptoms. In this ECHO cohort 15% of the children were exposed to significant neighborhood traffic, and there was a marked racial/ethnic disparity, with exposure rates being 39.5% in non-Hispanic Black children and 34.9% in Hispanic children. Similarly, there was a marked racial/ethnic disparity in the prevalence of past or current asthma/asthma-like symptoms.

The results from our statistical models indicate that the odds of having past and/or current asthma/asthma-like symptoms among children with reported high neighborhood traffic was 101% higher than the odds for children without reported high neighborhood traffic. These results are in similar direction as the Dutch Prevention and Incidence of Asthma and Mite Allergy (PIAMA) study, which examined associations between measured traffic-related air pollution and the development of asthma, allergy, and related symptoms in a prospective birth cohort [55]. The authors of the PIAMA study found air pollution measurements, specifically PM_2.5_ concentrations, were associated with increased incidence of asthma [OR = 1.28 (95% CI: 1.10, 1.49)], prevalence of asthma [OR = 1.26; 95% CI: 1.04. 1.51)], and prevalence of asthma symptoms [OR = 1.15 (95% CI, 1.02–1.28)] [55]. These associations were even stronger for children who remained at their birth residence. The authors stressed the need for more birth cohort studies, since the role of air pollution exposures in the development of childhood asthma, allergy, and related symptoms remains unclear.

There has been mounting evidence of a causal effect of TRAP exposures on asthma exacerbations [56]. Our findings are consistent with the results of a prospective study in Sweden (*n*~4000) which found positive associations between early life air pollution exposures (PM_10_ and NO_2_) and asthma exacerbations [57]. The Cincinnati birth cohort study (*n*~700) also detected associations between particulate air pollution exposure and wheezing during infancy [58]. Another study reported significant associations between traffic-related pollution and asthma exacerbations in children born to parents with atopic dermatitis and in those suffering from recurrent wheezing or asthma [59,60]. Our results add to the growing evidence on the importance of relevant windows of TRAP exposure.

A 2017 meta-analysis of 41 epidemiologic studies provided evidence for the hypothesis that childhood exposure to TRAP contributes to the development of asthma, and called for further studies [61]. A recent review, based on seven studies, concluded that TRAP exposures may be associated with transient and persistent asthma/wheezing phenotypes in children [56], and also stressed the need for more studies on these phenotypes. As children’s early life and current exposures to TRAP appears to play a key role in asthma development [18], carefully designed studies are needed to understand this relationship and identify critical windows for intervention.

Previous studies have assessed exposure to TRAP by directly measuring air pollutants, modelling, or indirectly by using distance to busy roads and/or self-reported traffic intensity [58,62,63,64]. A prospective cohort study of air pollution and respiratory health among children reported that incident asthma was positively associated with TRAP measured outside children’s residences for two weeks during the summer and winter [65]. Another birth cohort of racially diverse children (*n* = 24,608) reported an association between early-life mobile source models of air pollution and childhood asthma incidence [66]. A more recent studied showed that decreased ambient air pollutants in Southern California between 1993 and 2014 was associated with a lower incidence of childhood asthma [67].

In our study, we found that children of racial minority groups had a higher odds of asthma/asthma-like symptoms when compared to non-Hispanic White children which is in agreement with prior reports [68,69,70]. The odds of asthma/asthma-like symptoms for non-Hispanic Black and Hispanic children were 121% and 120% higher than the odds for non-Hispanic White children, respectively. This difference remained even after adjusting for mother’s education as a proxy for socioeconomic status [71]. A recent U.S. Centers for Disease Control and Prevention Report showed that asthma prevalence is more than twice as high among African American children (15.7%) and nearly twice as high in children of Puerto Rican (12.9%) descent compared with non-Hispanic White children (7.1%) [72,73].

Our results further showed that asthma/asthma-like symptoms were strongly associated with being male and having a positive family history of asthma. The odds of asthma/asthma-like symptoms for males was 30% higher than for females although this was not statistically significant; and a stratification analysis by sex revealed a 195% higher odds of reporting asthma/asthma-like symptoms for males in our cohort. Other studies have also reported increased risk of asthma and asthma-related symptoms in males, when compared to females [74,75,76]. In our study, children with asthma/asthma-like symptoms had more exposure to current secondhand smoke/environmental tobacco smoke (15.7% vs. 9.6%). However, after adjusting for demographic factors, this effect was no longer significant. We did not observe differences between asthma/asthma-like symptoms and the presence of cats and dogs either during the child’s first year or in the last 12 months. These results are similar to a prior cohort study [59].

We also found that obese children had 166% higher odds of having asthma/asthma-like symptoms than non-obese children. Additionally, children with play equipment in the home environment/backyard and those reporting the presence of neighborhood parks had 91% and 165% higher odds of having asthma/asthma-like symptoms than those without such amenities. The presence of traffic can negatively affect physical activity in the neighborhood [77,78] a key determinant of obesity [63]. Two longitudinal studies have reported the possible contribution of air pollution exposure to the development of obesity in children [63,79] and animal studies support these observations [80,81]. Parents may opt to have play equipment within the home environment, such as in a backyard, to allow for physical activity, and that may be of benefit, especially for families residing in urban areas [82]. However, parents may also need to consider potential TRAP exposures that could occur during outdoor physical activity.

This is the first study to report significant positive associations between asthma/asthma-like symptoms and play equipment in the home environment/backyard, even after adjusting for obesity. Children who had play equipment in their backyards and access to public parks had higher odds of asthma-like symptoms when obesity and prescribed asthma medication was added to our regression models. There may be several reasons for this. The effect of green spaces on wheezing/asthma symptoms may depend on the size of the space, amount of vegetation, and types of pollution present [83,84,85]. Studies have shown that regular physical activity has health benefits [86]. Additionally, other studies have shown that regular physical activity has health benefits for children when they are in low pollution rather than high pollution environments [87,88]. We found in the literature that environmental elements can alter the effects of air pollution. For instance, access to green space has physiologic benefits and tree cover can physically reduce levels of certain air pollutants, but these green spaces are not equal, and accessing them may be difficult due to heavy traffic [89]. While there is the possibility that playing in the backyard or public park exposes a child to TRAP, further research is needed to examine the possible interplay of TRAP with exercise, types of green spaces, and obesity in relation to asthma/asthma-like symptoms [90].

We also found that most children in our cohort (<90%) currently reside in urban areas, and for such children the odds were higher for reported asthma/asthma-like symptoms. Similarly, children without any reported prescribed asthma medication had higher odds of experiencing asthma/asthma-like symptoms. These findings indicate the need to expand childhood asthma management beyond preventing exposures to agents that directly cause allergic reactions to agents that cause a broad spectrum of non-specific, generalized inflammation, such as air pollution [91], particularly in urban areas which tend to have higher traffic-related air pollution.

Strengths of this study include the prospective longitudinal follow up of the children from mothers’ first trimester through up to 8 years of age and the racial/ethnic diversity of the cohort. The ISAAC questionnaire is a validated tool and the Pre-PAQ has also been reported to be both a valid and reliable measure of parental, family and neighborhood factors [49]. As such, we expect little misclassification, if any.

Our study also has several limitations. First, data are from maternal response to questionnaires and therefore subject to recall bias. However, we believe this bias was equally distributed among children with and without reported asthma/asthma-like symptoms, biasing effect estimates toward the null hypothesis. This is because the Pre-PAQ questions in this study assessed children’s physical activity and not specific environmental exposures. It is also important to note that some results might reflect a chance finding, given the large number of hypothesis tests and so a future confirmatory study may be necessary. An analysis comparing the current participants in this study with the participants who were eligible for this study revealed statistically significant differences in maternal race-ethnicity and education; however, participants were similar with respect to maternal age, child sex, gestational age at delivery, year of birth and birthweight (Table A1). We also believe that controlling for child’s race-ethnicity as well as maternal education mitigated any impact of potential selection bias and results remain informative.

In our study, there was an association between reported asthma/asthma-like symptoms and increased gestational age at delivery, but not for other factors such as mode of delivery, child’s use of antibiotic medication in the past year of child’s stay at the neonatal intensive care unit after birth. While early-term birth is a predictor of asthma, late term birth has also been associated with atopic dermatitis by 7 years of age [92]. Atopic dermatitis often precedes other allergic diseases such as asthma [93]. However, given the cross-sectional nature of our study, we are limited in discussing this topic. We concur with the strong need to identify alternatives for disease prevention [93]. Another limitation of our study is the lack of data on children’s diet and food allergies, which can lead to an increased risk of sensitization during childhood [94]. There may also be genetic differences in the children enrolled in this study and this may affect susceptibility to TRAP. Since our analysis is cross sectional, we are unable to determine whether exposure to TRAP preceded respiratory symptoms, or vice-versa, as such reverse causality is possible, as well as the possible time-related effects of obesity. Hence, we had limited power to fully delineate some potential associations. The current study is also limited because we do not have any pulmonary function data.

Finally, our exposure variable of interest, significant neighborhood traffic, was determined by questionnaire responses from mothers and we did not attempt to conduct air pollution exposure assessment outside their residences. It is important to note that the impact of early TRAP exposure on development of asthma, allergic rhinitis and aeroallergen sensitization in children remains unclear and the evidence from birth cohort studies appears to point to an association [95]. However, even with highly standardized outcomes and advanced exposure assessment methodology, evidence that TRAP increases the risk of these outcomes is inconsistent across birth cohort studies, particularly for allergic rhinitis and aeroallergen sensitization [96]. Long-term birth cohort studies with longitudinal data collection will be needed to confirm our findings. Future studies would benefit from greater standardization of exposure assessment, as well as harmonization of outcomes and confounders [61]. While we realize that actual measurements are preferable to a proxy such as reported traffic, we feel that such reports, especially used in conjunction with reports of the built environment, do provide useful information. A 2020 systematic review on the risk perception of air pollution reported that 31 out of 38 articles established a relationship between perception and measured pollution, even with variety of methodologies and population samples [97]. Clinicians should look closely at the contribution of an environmental factor such as traffic-related air pollution among patients who experience asthma/asthma-like symptoms to prevent deteriorating health. Additionally, public health professionals may also need to improve surveillance to help identify communities which may be at risk for increased TRAP concentrations.

## 5. Conclusions

Our findings provide evidence that there is a significant association between reported neighborhood traffic and asthma/asthma-like symptoms among children in the ECHO cohort. Minority status, male sex, the presence of play equipment within the home environment, public parks, family history of asthma, prescribed asthma medication, living in an urban area and obesity were also associated with asthma/asthma-like symptoms, highlighting the complexity of environmental and genetic interactions influencing lung function. Further studies are needed, since many children, particularly minority children at high risk of asthma, may live in close proximity to this source of environmental air pollution.

## Figures and Tables

**Table 1:** Comparison of the Environmental Influences on Child Health Outcomes Fetal Growth Study (ECHO-FGS) cohort by reported neighborhood traffic exposure.

Variable	All Children in Current Study (*n* = 855)%	Exposed to Neighborhood Traffic (*n* = 129)%	Unexposed to Neighborhood Traffic (*n* = 726)%	*p*-Value for χ^2^ or *t* Test
Child age in years, mean (SD)	6.9 (1.0)	6.7 (0.9)	6.9 (1.0)	0.09
Physician diagnosed asthma only (Asthma) [current or past]	13.9%	20.2%	12.8%	**0.03**
Wheezing or respiratory symptoms only (current or past)	19.1%	25.6%	17.9%	**0.04**
Primary Outcome: Asthma/asthma-like symptoms (current or past)	23.0%	33.3%	21.2%	**0.003**
Male child	51.2%	55.0%	50.6%	0.3
Race of child				
Non-Hispanic White (NHW)	31.1%	12.4%	34.4%	**<0.0001**
Non-Hispanic Black (NHB)	29.7%	39.5%	28.0%	
Hispanic	25.9%	34.9%	24.2%	
Asian	13.1%	13.2%	13.1%	
Mother Education				
Less than or up to high school	21.6%	34.9%	19.3%	**<0.0001**
More than high school	78.4%	65.1%	80.7%	
Family history of asthma	33.5%	34.9%	33.2%	0.9
Child’s BMI for age percentile				
Obese (BMI for age percentile ≥ 95)	11.2%	15.5%	10.5%	0.2
Child exposed to any secondhand smoke	11.0%	15.5%	10.2%	0.08
Household pets				
Cat at home during child’s first year and/or in the past 12 months	18.6%	16.3%	19.0%	0.5
Dog at home during child’s first year and/or in the past 12 months	44.7%	40.3%	45.5%	0.3
Pets (dog and/or cat) at home during child’s first year and/or in the past 12 months	52.8%	43.4%	54.4%	**0.02**
Inhalers and other asthma drug prescriptions (e.g., Pulmicort, Flovent, Singulair, Zyflo, Orapred, Advair, Symbicort, or Xolair)	17.2%	21.4%	16.5%	0.2
Hay fever or respiratory allergy	14.3%	12.4%	14.6%	0.5
Current residence in urban area	90.9%	92.1%	90.7%	0.6
Preschool-aged Children’s Physical Activity Questionnaire (Pre-PAQ) Q22 Variables [strongly agree or agree]				
Safety: It is safe for my child to play outdoors in my neighborhood (if supervised).	93.7%	79.1%	96.3%	**<0.0001**
Footpaths: There are usable footpaths on most of the streets in my local area.	78.0%	51.9%	82.6%	**<0.0001**
Walk: There are major barriers or dangers to walking with my child in my neighborhood that make it hard to get from place to place (e.g., major roads, railway lines, canals, storm water drains or rivers).	11.9%	45.7%	5.9%	**<0.0001**
Sufficient light: There are sufficient traffic lights or pedestrian crossings to make it safe to walk with my child around my neighborhood.	77.2%	48.8%	82.2%	**<0.0001**
Crime: The level of crime in my neighborhood makes it unsafe to go on walks with my child during the day.	9.5%	31.8%	5.5%	**<0.0001**
Shops: The local shop(s) are within easy walking distance of my home.	54.9%	54.3%	55.0%	1.0
Danger: There are dangers (e.g., dogs or undesirable people) in the local park(s) so I avoid taking my child there.	9.4%	32.6%	5.2%	**<0.0001**
Preschool-aged Children’s Physical Activity Questionnaire (Pre-PAQ) Q18 (please tick as many responses as apply) [Yes]				
Do you have access to any of the following facilities within your backyard or home environment?				
Play equipment (e.g., swing set, slide, climbing gym)	45.7%	31.8%	48.2%	**0.0006**
Pool or spa	19.8%	19.4%	19.8%	0.9
Area suitable to ride a tricycle, bike or scooter, etc.	78.0%	60.5%	81.1%	**<0.0001**
Preschool-aged Children’s Physical Activity Questionnaire (Pre-PAQ) Q21 Variables [Yes]				
Does your local neighborhood have the following places or facilities where your child can be play and be physically active? (please tick as many responses as apply)				
Open areas such as beaches, rivers, natural reserves	41.4%	29.5%	43.5%	**0.009**
Public park or oval	87.0%	76.7%	88.8%	**0.0002**
Playground	88.3%	79.1%	89.9%	**0.0003**
Public swimming pool	52.4%	44.2%	53.9%	0.1
Gym that offers programs for young children, e.g., kindergym, playgym, etc.	47.6%	35.7%	49.7%	**0.008**
Club that offers activities/sports for young children, e.g., soccer, dance, etc.	59.1%	49.6%	60.7%	**0.04**

*p* values in bold indicate statistical significance (*p* ≤ 0.05).

**Table 2:** Comparison of the Environmental Influences on Child Health Outcomes Fetal Growth Study (ECHO-FGS) cohort by primary outcome: asthma/asthma-like symptoms.

Variable	Asthma/Asthma-Like Symptoms (*n* = 197)%	No Asthma/Asthma-Like Symptoms (*n* = 658)%	*p*-Value for χ^2^ or *t* Test
Child age in years, mean (SD)	6.9 (1.0)	6.8 (1.0)	0.7
Male child	56.9%	49.5%	0.07
Race-ethnicity of child			
Non-Hispanic White (NHW)	19.3%	34.7%	**<0.0001**
Non-Hispanic Black (NHB)	40.1%	26.6%	
Hispanic	31.5%	24.2%	
Asian	8.6%	14.4%	
Mother Education			
Less than or up to high school	25.9%	20.4%	0.1
More than high school	74.1%	79.6%	
Family history of asthma	55.8%	26.8%	**<0.0001**
Child’s BMI for age percentile			
Obese (BMI for age percentile ≥ 95)	21.3%	8.2%	**<0.0001**
Child exposed to any second hand smoke	15.7%	9.6%	**0.02**
Household pets			
Cat at home during child’s first year and/or in the past 12 months	17.3%	19.0%	0.6
Dog at home during child’s first year and/or in the past 12 months	45.2%	44.5%	0.9
Pets (dog and/or cat) at home during child’s first year and/or in the past 12 months	51.8%	53.0%	0.8
Preschool-aged Children’s Physical Activity Questionnaire (Pre-PAQ) Q22 Variables [strongly agree or agree]			
Safety: It is safe for my child to play outdoors in my neighborhood (if supervised).	91.9%	94.2%	0.3
Footpaths: There are usable footpaths on most of the streets in my local area.	72.6%	79.6%	**0.04**
Walk: There are major barriers or dangers to walking with my child in my neighborhood that make it hard to get from place to place (e.g., major roads, railway lines, canals, storm water drains or rivers).	12.2%	11.9%	0.8
Neighborhood traffic: There is so much traffic along the streets that it makes it difficult or dangerous to walk with my child in my neighborhood.	21.8%	13.1%	**0.003**
Sufficient light: There are sufficient traffic lights or pedestrian crossings to make it safe to walk with my child around my neighborhood.	71.6%	78.9%	0.06
Crime: The level of crime in my neighborhood makes it unsafe to go on walks with my child during the day.	11.7%	8.8%	0.2
Shops: The local shop(s) are within easy walking distance of my home.	56.9%	54.3%	0.6
Danger: There are dangers (e.g., dogs or undesirable people) in the local park(s) so I avoid taking my child there.	13.2%	8.2%	**0.04**
Preschool-aged Children’s Physical Activity Questionnaire (Pre-PAQ) Q18 (please tick as many responses as apply) [ Yes]			
Do you have access to any of the following facilities within your backyard or home environment?			
Play equipment (e.g., swing set, slide, climbing gym)	48.7%	44.8%	0.3
Pool or spa	21.8%	19.2%	0.5
Area suitable to ride a tricycle, bike or scooter, etc.	77.2%	78.3%	0.9
Preschool-aged Children’s Physical Activity Questionnaire (Pre-PAQ) Q21 Variables [Yes]			
Does your local neighborhood have the following places or facilities where your child can be play and be physically active? (please tick as many responses as apply)			
Open areas such as beaches, rivers, natural reserves	40.1%	41.8%	0.7
Public park or oval	90.4%	86.0%	**0.03**
Playground	86.3%	88.9%	0.6
Public swimming pool	53.8%	52.0%	0.5
Gym that offers programs for young children, e.g., kindergym, playgym, etc.	48.7%	47.3%	0.4
Club that offers activities/sports for young children, e.g., soccer, dance, etc.	58.9%	59.1%	0.4

*p* values in bold indicate statistical significance (*p* ≤ 0.05).

**Table 3:** Odds ratios with 95% confidence limits for the primary outcome: asthma/asthma-like symptoms in relationship to demographics, home and neighborhood characteristics of the Environmental Influences on Child Health Outcomes Fetal Growth Study (ECHO-FGS) cohort.

	Model 1 (*n* = 835)	Model 2 (*n* = 825)	Model 3 (*n* = 817)	Model 4 (*n* = 738)	Model 5 (*n* = 733)
Male child	**1.52 (1.08, 2.15)**	**1.57 (1.11, 2.23)**	**1.54 (1.08, 2.19)**	**1.53 (1.06, 2.22)**	1.30 (0.83, 2.04)
Age of child	1.20 (0.98, 1.46)	1.20 (0.99, 1.47)	1.21 (0.99, 1.47)	1.18 (0.95, 1.45)	1.21 (0.93, 1.56)
Race-Ethnic of child					
Non-Hispanic White (NHW)	1.00	1.00	1.00	1.00	1.00
Non-Hispanic Black (NHB)	**2.55 (1.52, 4.29)**	**2.74 (1.61, 4.67)**	**2.68 (1.56, 4.59)**	**2.53 (1.43, 4.50)**	**2.21 (1.10, 4.47)**
Hispanic	**2.39 (1.44, 3.98)**	**2.62 (1.55, 4.42)**	**2.56 (1.52, 4.34)**	**2.25 (1.28, 3.94)**	**2.20 (1.11, 4.32)**
Asian	1.41 (0.71, 2.80)	1.58 (0.79, 3.18)	1.50 (0.74, 3.01)	1.40 (0.66, 2.99)	1.37 (0.55, 3.37)
Mother Education	0.93 (0.61, 1.41)	0.91 (0.59, 1.39)	0.97 (0.63, 1.50)	1.08 (0.69, 1.70)	1.17 (0.67, 2.03)
Family History of asthma	**3.32 (2.34, 4.70)**	**3.38 (2.38, 4.81)**	**3.24 (2.27, 4.63)**	**3.19 (2.20, 4.64)**	**1.99 (1.26, 3.15)**
Neighborhood Traffic	**1.59 (1.02, 2.48)**	**1.77 (1.13, 2.77)**	**1.93 (1.22, 3.06)**	**1.78 (1.10, 2.88)**	**2.01 (1.12, 3.62)**
Play Equipment	----	**1.53 (1.07, 2.19)**	**1.44 (1.00, 2.07)**	**1.60 (1.09, 2.35)**	**1.91 (1.19, 3.09)**
Public Park	----	----	**2.02 (1.07, 3.79)**	1.86 (0.98, 3.54)	**2.65 (1.14, 6.15)**
Obese	----	----	----	**2.54 (1.55, 4.17)**	**2.66 (1.44, 4.92)**
Asthma medication	----	----	----	----	**25.07 (14.81, 42.45)**

Odds ratios in bold indicate statistical significance (*p* ≤ 0.05).

## Data Availability

Data contains personal health information and cannot be made publicly available. Data can be available from the authors upon reasonable request, with permission of NICHD and with establishment of required data use agreements. Any data inquiries are referred to the PI (huntke@musc.edu).

## Appendix A

**Table A1:** Comparison of the current cohort for Environmental Influences on Child Health Outcomes Fetal Growth Study (ECHO-FGS) compared to the participants eligible for the entire study.

Variable	Participants in Current Study (*n* = 855)	Participants Eligible for ECHO Study (*n* = 2373)	*p*-Value for χ^2^ or *t* Test
Mother’s age, mean (SD) in years	28.6 (5.6)	28.3 (5.5)	0.2
Mother’s education			**<0.0001**
Less than or up to high school	21.6%	29.6%	
More than high school	78.4%	70.4%	
Mother’s race			**<0.0001**
Non-Hispanic White (NHW)	34.0%	26.3%	
Non-Hispanic Black (NHB)	29.6%	27.8%	
Hispanic	22.8%	28.7%	
Asian	13.6%	17.2%	
Male child	51.2%	50.8%	0.8
Child’s median year of birth (range)	2011 (2010 to 2013)	2011 (2009 to 2013)	0.5
Child’s gestational age at delivery, mean (SD) in weeks	39.2 (1.5)	39.1 (1.9)	0.6
Child’s birthweight, mean (SD) in kg	3.2 (0.5)	3.3 (0.5)	0.06

*p* values in bold indicate statistical significance (*p* ≤ 0.05).
